# Risk factors in piano playing techniques for developing playing-related musculoskeletal disorders and focal dystonia: a scoping review

**DOI:** 10.13075/ijomeh.1896.02670

**Published:** 2026

**Authors:** Anita Maria Tummolo, Marcello Di Pumpo, Pier Carlo Battain, Marina Ramella, Rosa Maria Converti

**Affiliations:** 1 University of Milan, Rehabilitation Medicine for Stage Artists, Milan, Italy; 2 Catholic University of the Sacred Heart, Department of Life Sciences and Public Health, Rome, Italy; 3 IRCCS Santa Maria Nascente – Don Carlo Gnocchi Foundation, Milan, Italy

**Keywords:** rehabilitation, prevention, focal dystonia, PRMD, piano, pianists

## Abstract

This scoping review aims to explore risk factors related to piano technique that contribute to the onset of playing-related musculoskeletal disorders (PRMDs) and focal dystonia in pianists, providing a comprehensive synthesis of the current evidence. A scoping review was conducted using the PubMed database in September–October 2024. Eligible studies included professional pianists or students and investigated technical, ergonomic, demographic, or psychological risk factors associated with PRMDs or focal dystonia. Both qualitative and quantitative peer-reviewed studies were considered. Screening was performed in double-blind, and data were extracted regarding practice habits, symptoms, anatomical areas affected, and hypothesized risk factors. Seventeen studies met inclusion criteria. Key risk factors for PRMDs included small hand size, repetitive motion, extended practice sessions without adequate rest, and suboptimal ergonomic setups. The most affected anatomical regions were the wrists, shoulders, neck, and back. Psychological stress and performance anxiety were frequently associated with physical symptoms. For focal dystonia, repetitive technical exercises, poor neuromuscular recovery, early specialization, and stress were identified as critical contributors. Lack of awareness and education on injury prevention was a consistent theme, particularly among students. The review highlights the multifactorial etiology of PRMDs and focal dystonia in pianists. Effective prevention requires ergonomic adjustments, structured practice routines, repertoire adaptation, and health education. Piano pedagogy should integrate physical and psychological strategies to support long-term performance health. Standardized definitions and longitudinal research are needed to improve understanding and guide evidence-based interventions.

## Highlights

- A small hand size is a risk factor for the development of playing-related musculoskeletal disorders (PRMDs) in pianists.
- Stretching and warming up are protective factors against the development of PRMD.
- Psychological stress is a risk factor for the development of focal dystonia.

## INTRODUCTION

Playing-related musculoskeletal disorders (PRMDs) and focal dystonia represent significant occupational hazards for pianists, impacting their ability to perform and often leading to career limitations. Numerous terms have been used to describe musicians' musculoskeletal disorders, including “overuse syndrome” [1,2], “repetitive strain injury” [3] and “cumulative trauma disorder” [4,5]. Lack of consensus regarding terminology has led to confusion in this field [6,7]. A qualitative research by Zaza et al. [8] has derived the following operational definition of PRMDs: “(…) pain, weakness, lack of control, numbness, tingling, or other symptoms that interfere with your ability to play your instrument at the level you are accustomed to.” This operational definition of PRMD was validated as an outcome measure in a risk factor study of musicians [8] and it will be used in this scoping review.

Therefore, PRMDs are characterized by pain, weakness, or stiffness that interferes with the ability to play an instrument at the desired level, while focal dystonia involves involuntary muscle contractions that disrupt fine motor control. These conditions are highly prevalent among musicians, with studies reporting PRMD prevalence rates ranging 38–89% depending on the population and assessment methods [9,10].

For pianists, the physical demands of playing, such as repetitive movements, prolonged practice sessions, and suboptimal ergonomic setups, are known contributors to PRMDs [11]. Smaller hand size and anthropometric mismatches with piano key dimensions have also been identified as risk factors, especially among female players [10]. Practice habits, including the duration and frequency of breaks, play a crucial role in mitigating or exacerbating these risks [12].

Despite the growing awareness of these disorders, there is a lack of standardized preventative measures or universally accepted guidelines to minimize their occurrence. This scoping review aims to synthesize the existing literature to identify potential risk factors related to piano playing technique that may contribute to the development of PRMDs and focal dystonia. This work is particularly important as there is currently no scoping review that has summarized the existing literature regarding the onset of PRMDs specifically in pianists.

By analyzing the findings from different populations and methodologies, this study seeks to hypothesize preventive strategies and guide future research efforts in this critical area of musician health.

## METHODS

A scoping review was conducted to identify potential risk factors in piano-playing techniques associated with the development of PRMD or focal dystonia.

The inclusion criteria encompassed: studies involving professional pianists or piano students reporting on risk factors associated with PRMD or focal dystonia; articles presenting quantitative or qualitative data on practice habits, ergonomics, demographics factors; peer-reviewed publications written in English, made available in the full version, that included interventional studies, observational studies, scoping reviews, systematic review and meta-analysis. No restriction for time of publication were defined.

The exclusion criteria encompassed: studies not relevant to the research objective, studies that did not report primary data relating to risk factors in piano players and research that focused exclusively on treatment or rehabilitation interventions, neglecting the analysis of risk factors, study design different from what defined in the inclusion criteria.

A bibliographic database search was conducted in September and October 2024. To search the literature, the database of the National Library of Medicine, i.e., PubMed, was queried, as it represents the most complete, authoritative and specialized source in the health and medical sciences. The decision to use this specific database was motivated by the need to access peer-reviewed studies of high scientific quality, and in consideration of the very specialized topic across the musical and medical domain expanding to multiple may introduce studies from areas (e.g., music education, psychology) of less relevance to the question under consideration.

**Figure 1.**
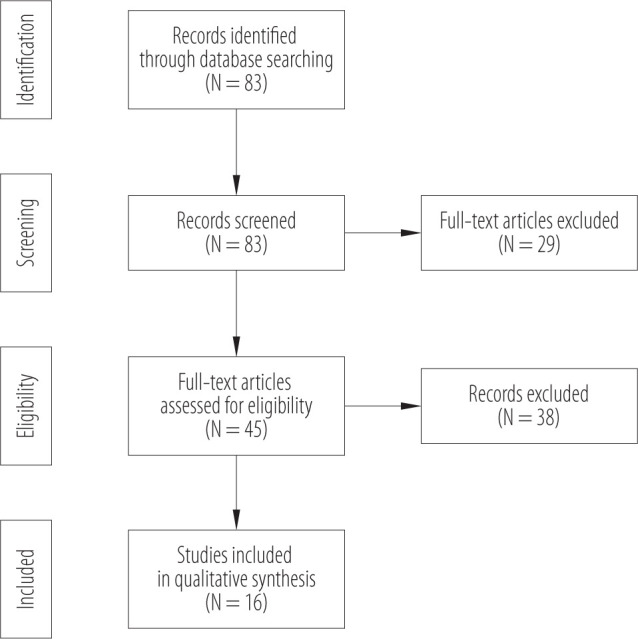
PRISMA 2020 flow diagram of the study selection process for the scoping review on piano-technique-related risk factors for playing-related musculoskeletal disorders (PRMDs) and focal dystonia in pianists, PubMed search, September–October 2024

The search string was produced interpolating the keywords “piano,” “pianist,” “playing-related musculoskeletal disorders,” “focal dystonia,” and is available as Figure 1 allowing the inclusion of and considering primary research articles as study type.

The studies resulting from the search were subjected to a title-abstract and subsequent full-text screening carried out in double-blind by 2 members of the research team, with conflicts resolution performed by a third research member, in order to guarantee the objectivity and coherence of the process.

For each of the selected studies, data related to demographic characteristics, piano practice habits, reported symptoms, affected anatomical regions and hypothesized risk factors were collected. Information on the study design, the characteristics of the population and the outcomes observed were also recorded, with the aim of ensuring a summary that was as complete and accurate as possible.

## RESULTS

Seventeen studies were included in the final analysis. The PRISMA [13] flowchart presents the details of the study selection (Figure 1).

The studies varied in their methodological approach, ranging from systematic reviews and observational crosssectional studies to experimental and qualitative research (Table 1).

**Table 1:** Characteristics and study designs of the 16 articles included in the scoping review on piano-technique-related risk factors for playing-related musculoskeletal disorders (PRMDs) and focal dystonia in professional pianists and piano students, PubMed search, September–October 2024

Study	Study design
Baeyens et al., 2022 [21]	experimental study; examined upper trapezius muscle activity in conservatory piano students in relation to rehearsal time and repertoire speed
Bragge et al., 2006 [10]	systematic review; synthesized prevalence and risk factors associated with playing-related musculoskeletal disorders in pianists
Bruno et al., 2008 [9]	cross-sectional observational study; assessed disabling musculoskeletal disorders in young and adult classical piano students
Chi et al., 2020 [11]	systematic review; analyzed existing literature on ergonomics in violin and piano playing
Clijsen et al., 2023 [25]	observational study; measured intramuscular oxygenation and extensor carpi radialis brevis muscle activity during piano performance
Degrave et al., 2020 [22]	electromyographic experimental study; investigated upper limb muscle activity during isolated piano keystrokes
Frucht, 2008 [15]	narrative review; provided a clinical approach to focal task-specific dystonia of musicians' hands
Linari-Melfi et al., 2011 [16]	cross-sectional observational study; investigated deep tissue hypersensitivity to pressure pain in professional pianists with insidious mechanical neck pain
Ling et al., 2016 [18]	cross-sectional observational study; investigated the knowledge of playing-related musculoskeletal disorders among classical piano students in Malaysian tertiary institutions
Ling et al., 2018 [12]	cross-sectional observational study; examined the prevalence and associated risk factors of musculoskeletal disorders among classical piano students
McKechnie and Jacobs, 2011 [14]	observational study; explored physical and environmental factors contributing to music-related injuries among children
Thio-Pera et al., 2022 [17]	experimental study; compared forearm muscle excitation levels in different professional piano players using electromyography
Xiaoyu, 2024 [24]	qualitative phenomenological study; explored risk factors and coping strategies for PRMDs in tertiary-level student pianists
Yoshie et al., 2009 [23]	experimental study; examined music performance anxiety in skilled pianists, assessing subjective, autonomic, and electromyographic responses
Yuen et al., 2021 [20]	cross-sectional observational study with photogrammetry; analyzed upper-body musculoskeletal disorders among piano and non-piano players using photogrammetry
Zhao et al., 2024 [19]	cross-sectional observational study; evaluated the prevalence, risk factors, and preventive interventions for PRMDs among Chinese conservatory piano students

The following main themes as key drivers for the analysis: demographic characteristics, anthropometric features, ergonomic issues, biomechanical aspects of piano technique and choice of repertoire, reported symptoms and affected regions, specific risk factors for focal dystonia (Table 2).

**Table 2:** Key findings of included studies, organized by category of risk factor (demographic, anthropometric, ergonomic, biomechanical/technical, symptom-related, and focal-dystonia-specific) for playing-related musculoskeletal disorders (PRMDs) and focal dystonia in professional pianists and piano students, PubMed search, September–October 2024

Category	Key findings
Demographic characteristics	– gender influences PRMD prevalence – higher incidence in females [12] – children face physical demands and poor ergonomics [23]
Anthropometric features	– small hand size linked to higher PRMD risk due to biomechanical strain [9,11,14,15]
Ergonomic issues	– practice >3 h/day or >20 h/week increases PRMD risk – lack of breaks and insufficient stretching also linked to higher incidence [9,17,19]
Biomechanical technique and repertoire	– struck key touch increases upper limb strain [20] – fast and virtuosic repertoire increases muscle activity and fatigue [10,11,19]
Reported symptoms and affected regions	– common symptoms: pain, fatigue, stiffness; affected regions: wrist, shoulder, neck, back – stress contributes to maladaptive behaviors [9,11,21]
Focal dystonia risk factors	– linked to repetition, small hand size, perfectionism, lack of breaks, and early specialization [9,11–13]

### Demographic characteristics

Most studies reported age and gender, with 2 reporting only age [10,11] and 1 only gender [13]. Particularly gender was associated with PRMD prevalence, with a higher incidence reported among female pianists – possibly due to smaller average hand size and related ergonomic challenges [12].

McKechnie et al. [14] investigated physical and environmental risk factors in children, emphasizing that immature musculoskeletal systems combined with high physical demands can predispose young musicians to PRMD. Key issues include:

– instrument size mismatch, where oversized pianos or poorly adjusted seating result in unnatural postures and strain,
– repetitive movement without breaks, increasing the risk of overuse injuries,
– environmental factors, such as poor lighting or seating, that contribute to discomfort during practice.

### Anthropometric features

Four studies [9,12,15,16] highlighted small hand size as a major risk factor for PRMD. The standard keyboard dimensions often force pianists with smaller hands into biomechanically disadvantageous positions, leading to increased strain. These findings emphasized the importance of considering anthropometric characteristics in ergonomic assessments of piano playing.

### Ergonomic issues

Seven studies [9,12,17–20] addressed practice habits and ergonomic risk factors:

– practice duration – daily sessions >3 h and weekly practice >20 h were associated with higher PRMD prevalence [9,16];
– breaks during practice – inadequate rest increases fatigue and injury risk; regular breaks were shown to support muscle recovery and reduce PRMD incidence [12,17,19];
– stretching and warm-ups – regular use of these strategies improves flexibility and reduces tension – pianists who performed stretching exercises before and after practice reported fewer symptoms [9]; additionally, Chi et al. [11] found these routines psychologically beneficial, aiding focus and reducing performance anxiety.

Despite widespread recognition of the value of warm-up and stretching routines in injury prevention [9,11], their application among pianists remains inconsistent. This inconsistency is largely due to a lack of formal education on ergonomic practices during piano instruction. Educators and health professionals must work together to integrate these preventive strategies into daily practice routines, especially at early stages of musical training. For young pianists, ensuring proper instrument size, seating height, and keyboard accessibility is essential [24]. Incorporating structured breaks into lessons, fostering an awareness of good posture, and teaching students to recognize early signs of strain can prevent injuries before they become chronic. Parents and teachers must collaborate to create environments that encourage safe, sustainable music-making from the outset.

A notable finding across several studies is the general lack of awareness among student pianists regarding the risks and signs of PRMD [17]. This suggests an urgent need for curriculum-based health education initiatives, ideally integrated into early piano training. Programs should cover topics such as optimal practice durations, importance of breaks, stretching techniques, and body mechanics. Equipping young musicians with this knowledge not only supports physical well-being but also empowers them to take proactive roles in their health. Moreover, incorporating injury prevention into teaching certifications could ensure that future piano educators are equipped to address these issues in their pedagogy. Raising awareness at all levels of instruction – from private lessons to conservatory education – can create a cultural shift in how musicians approach their physical health. This preventative approach has the potential to reduce the high prevalence of PRMD in pianists.

### Biomechanical aspects of piano technique and choice of repertoire

Four studies [10,11,20,21] examined technical playing styles and their physical consequences. A key distinction was made between the “struck” and “pressed” key touch techniques. The struck touch – characterized by fast, forceful motion – elicited greater muscle activation in the upper limbs compared to the more controlled pressed touch [20], potentially increasing fatigue and injury risk.

Repertoire selection also plays a central role. Baeyens et al. [21] found that fast-paced, technically demanding pieces raised trapezius muscle activity in conservatory students, leading to increased fatigue. Prolonged exposure to such repertoire without recovery was linked to a greater risk of PRMD.

Other studies supported this conclusion. Bragge et al. [10] noted that repetitive, high-speed passages can disproportionately affect pianists with small hands or lower muscular endurance. Chi et al. [11] emphasized the physical and psychological toll of virtuosic repertoire, linking it to fatigue, overuse injuries, and performance anxiety.

The implications for piano pedagogy are profound. Educators should not only focus on musical interpretation and technical precision, but also on physical health education. Training should include modules on ergonomics, movement awareness, and physical self-care strategies. By teaching students to recognize early warning signs of strain and to adopt injury-prevention strategies, teachers can help mitigate long-term physical problems. Structured practice routines that include short breaks every 30–45 min have been shown to decrease fatigue. Emphasis should also be placed on quality of practice over quantity. Furthermore, piano design innovations such as adjustable keyboards that accommodate hand size differences could become instrumental in reducing strain for smaller-handed pianists. The selection of repertoire is another area that requires careful attention [25]. Assigning pieces that are appropriate to the student's hand span and muscular endurance is essential to avoid unnecessary physical challenges [10,120]. Teachers should rotate between complex and less demanding works to allow recovery while still maintaining skill development. Ultimately, pedagogical approaches must be individualized and holistic, fostering technical excellence without compromising physical well-being.

### Reported symptoms and affected regions

Five studies [2,9–11,22] provided detailed information on the symptoms and anatomical areas affected by PRMD in pianists. The most frequently reported symptoms included pain, fatigue, and stiffness. The most involved regions were the shoulders, back, neck, and wrists.

Bruno et al. [9] observed that wrist strain was particularly evident during rapid passages and repetitive technical exercises, where the demand for fine motor control leads to localized muscle fatigue. Similarly, Chi et al. [11] and Yoshie et al. [23] reported that shoulder and neck discomfort stemmed from the sustained elevation and stabilization of the arms required during prolonged playing sessions.

Yoshie et al. [23] also highlighted the interplay between psychological stress and physical strain, indicating that performance anxiety can lead to maladaptive motor behaviors, which in turn elevate the risk of injury. Back pain, especially in the thoracic and lumbar spine, was attributed to extended seated postures and insufficient core support [10,23]. These findings reinforce the multifactorial nature of PRMD, where posture, technique, and psychological factors intersect to affect musicians' health.

### Specific risk factors for focal dystonia

Five studies [9,10–13] explored focal dystonia, identifying the following risk factors:

– repetitive technical movements – rapid finger exercises and forceful keystrokes were shown to overstimulate neuromuscular pathways, disrupting motor control [11,13],
– small hand size and biomechanical mismatch – pianists with small hands performing repertoire designed for larger spans may develop maladaptive movement strategies, increasing dystonia risk [10],
– psychological stress – high levels of perfectionism and performance anxiety can interfere with automatic motor control, leading to dystonic symptoms [12],
– insufficient rest – continuous practice without breaks may impair neuromuscular recovery and motor learning [11],
– early specialization – Bruno et al. [9] suggested that intensive practice during childhood could establish rigid motor patterns, making pianists more susceptible to dystonia later in life.

Prevention of focal dystonia requires varied technical exercises and structured practice with breaks [9,11,12]. Stress management and mindfulness help reduce perfectionism-related tension. Training programs should include education on dystonia risks and strategies, including body awareness, warm-ups, and ergonomic adjustments.

Although most research on PRMD focuses on mechanical and behavioral causes, some evidence points to a possible genetic or neurological predisposition to certain disorders, notably focal dystonia [13]. While focal dystonia is relatively rare, it is particularly debilitating due to its impact on fine motor control. Familial clustering observed in some studies indicates that genetic factors may contribute to its onset, possibly through inherited variations in motor control or neurological wiring. Understanding these predispositions could be crucial for early detection, especially in young pianists from families with a history of dystonia or related conditions. Though not yet conclusive, this area of research highlights the need for interdisciplinary approaches that integrate genetics, neurology, and music performance science to build comprehensive prevention strategies.

## CONCLUSIONS

Several limitations temper the strength of these findings. A notable challenge is the inconsistency in how PRMD is defined across studies, which complicates efforts to synthesize results and draw definitive conclusions. Additionally, age and gender biases are evident in many studies, with limited analysis of diverse age groups and insufficient gender-specific evaluations. This gap restricts the generalizability of findings and hinders the development of tailored interventions. Self-reported data on symptoms and practice habits further weakens the reliability of the evidence, introducing potential inaccuracies and subjective biases. Lastly, the absence of longitudinal studies is a critical limitation, as it prevents establishing causality between identified risk factors and the onset of PRMD, leaving significant gaps in understanding the temporal dynamics of these conditions.

In conclusion, the findings underscore the importance of a holistic approach to PRMD prevention, combining ergonomic adjustments, education, and behavioral strategies. By integrating these insights into practice and pedagogy, the piano-playing community can work toward reducing the burden of PRMD and fostering sustainable, injury-free performance careers.

In sum, while the evidence base provides meaningful insights into PRMD risk factors, addressing its limitations through standardized definitions, longitudinal studies, and diverse participant analyses will be crucial for advancing the field. Furthermore, a systematic review of risk factors and an expansion of the databases queried could be useful to further understand the mechanisms underlying the onset of PRMD in pianists. Integrating the findings into educational and practical strategies can foster a healthier playing environment, ensuring that pianists can sustain their careers without compromising their physical well-being.
